# Ameliorating Effects of *Bacillus subtilis* ANSB060 on Growth Performance, Antioxidant Functions, and Aflatoxin Residues in Ducks Fed Diets Contaminated with Aflatoxins

**DOI:** 10.3390/toxins9010001

**Published:** 2016-12-22

**Authors:** Liyuan Zhang, Qiugang Ma, Shanshan Ma, Jianyun Zhang, Ru Jia, Cheng Ji, Lihong Zhao

**Affiliations:** 1 State Key Laboratory of Animal Nutrition, College of Animal Science and Technology, China Agricultural University, Beijing 100193, China; zhangliyuan619@163.com (L.Z.); maqiugang@cau.edu.cn (Q.M.); jyzhang@cau.edu.cn (J.Z.); jicheng@cau.edu.cn (C.J.); 2 Heilongjiang Animal Science Institute, Qiqihar 161005, China; mashanshan33@foxmail.com; 3 College of Life Science, Shanxi University, Taiyuan 030006, China; jiaru.bjdk071@163.com

**Keywords:** aflatoxin B_1_, aflatoxin biodegradation preparation, *Bacillus subtilis* ANSB060, ameliorating effects, growth performance, antioxidant function, residue

## Abstract

*Bacillus subtilis* ANSB060 isolated from fish gut is very effective in detoxifying aflatoxins in feed and feed ingredients. The purpose of this research was to investigate the effects of *B. subtilis* ANSB060 on growth performance, body antioxidant functions, and aflatoxin residues in ducks fed moldy maize naturally contaminated with aflatoxins. A total of 1500 18-d-old male Cherry Valley ducks with similar body weight were randomly assigned to five treatments with six replicates of 50 ducks per repeat. The experiment design consisted of five dietary treatments labeled as C0 (basal diet containing 60% normal maize), M0 (basal diet containing 60% moldy maize contaminated with aflatoxins substituted for normal maize), M500, M1000, and M2000 (M0 +500, 1000 or 2000 g/t aflatoxin biodegradation preparation mainly consisted of *B. subtilis* ANSB060). The results showed that ducks fed 22.44 ± 2.46 μg/kg of AFB_1_ (M0) exhibited a decreasing tendency in average daily gain (ADG) and total superoxide dismutase (T-SOD) activity in serum, and T-SOD and glutathione peroxidase (GSH-Px) activities in the liver significantly decreased along with the appearance of AFB_1_ and AFM_1_ compared with those in Group C0. The supplementation of *B. subtilis* ANSB060 into aflatoxin-contaminated diets increased the ADG of ducks (*p* > 0.05), significantly improved antioxidant enzyme activities, and reduced aflatoxin accumulation in duck liver. In conclusion, *Bacillus subtilis* ANSB060 in diets showed an ameliorating effect to duck aflatoxicosis and may be a promising feed additive.

## 1. Introduction

Aflatoxins (AF) are secondary toxic metabolites produced by certain fungi belonging to the genus *Aspergillus* and can occur as natural contaminants of poultry food [1,2,3,4,5]. The principal aflatoxins commonly found in feedstuffs are aflatoxin B_1_ (AFB_1_), aflatoxin B_2_ (AFB_2_), aflatoxin G_1_ (AFG_1_), and aflatoxin G_2_ (AFG_2_) [6]. AFB_1_ has the highest toxic potency and is classified as a Group I carcinogen by the International Agency for Research on Cancer (IARC, International Agency for Research on Cancer, 1993). After being ingested by animals, AFB_1_ is converted to AFB_1_-8, 9-epoxide in the liver with a reaction catalyzed by cytochrome P-450 isozymes, forming adducts with the guanine base of DNA, thus resulting in the toxicity of AFB_1_ [7,8].

Feeds contaminated with AFB_1_ can result in aflatoxicosis in poultry [9,10]. Many studies have found that feeding grains contaminated with AFB_1_, either naturally or purified, can reduce growth performance and immunity ability, alter intestinal morphology and blood biochemistry parameters, and damage liver and kidney tissues in broilers [11,12]. Additionally, residues of aflatoxins and their metabolites may be present in meat and other products of animals fed rations contaminated with aflatoxins and potentially result in health problems in human.

It is known that aflatoxins are difficult or impossible to be removed completely from plants and living organisms, as they can alter the chemical structure of mycotoxins as part of their defense against xenobiotics [13]. Researchers have tried for decades to find effective strategies regarding mycotoxin detoxification. The multifaceted approaches in previous reports could simply be categorized into physical, chemical, and biological methods. A physical method is using non-nutritive absorptive materials in animal diets to reduce the toxin absorption from the gastrointestinal tract of chickens [14,15]. A study showed that Blasticidin A (BcA), an antibiotic produced by Streptomyces, can control aflatoxin production by inhibiting protein synthesis [16]. Recently, the interest of researchers has turned to the biological detoxification of mycotoxins using microorganisms or enzymatic preparation for their specificity, efficiency, and simplicity [17,18,19,20,21]. Some microbes such as *Armillariella tabescens* [22], *Rhodococcus erythropolis* [23], and *Myxococcus fulvus* [24] have been reported to possess the ability to degrade aflatoxins. However, most of the microorganisms are not allowed to be applied in animal feed under the supervision of the U.S. Food and Drug Administration (FDA), the Association of American Feed Control Officials (AAFCO), and the Direct of Ministry of Agriculture, People’s Republic of China. Based on this concern, we have isolated a strain of *Bacillus subtilis* ANSB060 from fish gut that possesses a strong ability to detoxify aflatoxins. We also have confirmed that this strain can inhibit the growth of pathogens and resist unfavorable conditions within simulated gut environments [25]. Since the nutritional and pharmaceutical use of *Bacillus subtilis* is generally recognized as safe (GRAS), the Ministry of Agriculture of the People’s Republic of China has permitted *B. subtilis* for use in animal feed. Ma et al. (2012) have shown that the addition of *B. subtilis* ANSB060 can enhance the activity of antioxidant enzymes and recover the protein synthesis in the livers of laying hens [26], and Fan et al. (2013, 2015) verified its positive effects of detoxifying aflatoxins on broilers [27,28].

Ducks are considered to be highly susceptible because they are unable to efficiently metabolize aflatoxins [10]. A study examining the effects of AFB_1_ on liver lesions in poultry reported that ducks generated hepatic lesions on alternate days per os 15 μg AFB_1_ [29]. Other literature on ducks fed diets naturally contaminated with AFB_1_ is also available [30,31]. However, to the best of our knowledge, little information on biological detoxification methods dealing with duck diets contaminated with AFB_1_ has been found. China has a large consumer of duck products in the world (FAO, 2003), so it is of great importance to study the effects of aflatoxins on ducks and develop solutions to detoxify aflatoxins to ensure the qualities of duck products. We have investigated the toxic effects of aflatoxins and the efficacy of *Bacillus subtilis* ANSB060 for the amelioration of aflatoxicosis in broiler chickens. Whether low levels of aflatoxins can affect the growth and antioxidant functions of ducks and which level of aflatoxins in diets can result in residues in duck tissues is still under consideration. Thus, certifying the effects of low levels of aflatoxins on ducks and finding a reasonable method to degrade aflatoxins in diets is important. Therefore, the objective of this study was to investigate the protective effects of aflatoxin biodegradation preparation (*B. subtilis* ANSB060) on growth performance, body antioxidant functions, and aflatoxin residues in ducks fed maize naturally contaminated with AFB_1_.

## 2. Results

### 2.1. Growth Performance

The effects of *B. subtilis* ANSB060 on growth performance of Cherry Valley ducks fed moldy maize naturally contaminated with aflatoxins are presented in Table 1. There were no significant difference on ADG and ADFI among ducks fed basal diet without AFB_1_ (C0), a moldy diet naturally contaminated with AFB_1_ (M0), and diets (M500, M1000, and M2000) naturally contaminated with AFB_1_ mixed with *B. subtilis* ANSB060 during the experimental phase (*p* > 0.05). However, birds fed a diet with 22.44 ± 2.46 μg/kg of AFB_1_ (M0) had a relatively smaller ADG (*p* > 0.05) compared to those fed the basal diet without AFB_1_ (C0), while ducks fed 22.44 ± 2.46 μg/kg of AFB_1_ with *B. subtilis* ANSB060 exhibited relatively higher ADG (*p* > 0.05) compared to those in Group M0. The feed/gain ratio (F:G) of ducks was significantly affected by the dietary aflatoxins and *B. subtilis* ANSB060 during the experimental phase (*p* < 0.05). The F:G of birds in Group M1000 was markedly decreased compared with that in Group M0 (*p* < 0.05), implying that *B. subtilis* ANSB060 in diets might relieve the negative effects of aflatoxion on birds and improve the feed efficiency of ducks.

### 2.2. Serum Antioxidant Indices

The effects of *B. subtilis* ANSB060 on the serum antioxidant indices of ducks exposed to aflatoxins are shown in Table 2. The total superoxide dismutase (T-SOD) activity of the M0 group was decreased by the dietary aflatoxins compared to that in Group C0 during the experimental phase (*p* < 0.05). However, the addition of *B. subtilis* ANSB060 into moldy diets significantly improved the activity of serum T-SOD in the Group M500, M1000 and M2000 (*p* < 0.05), maintaining it in a normal status showed in Group C0. Though the glutathione peroxidase (GSH-Px) activity and malondiadehyde (MDA) content were not different among all groups (*p* > 0.05), Group M0 had the lowest GSH-Px activity and the highest MDA content, and the dietary *B. subtilis* ANSB060 could raise the activity of GSH-Px and decrease MDA concentration, keeping them to the normal level.

### 2.3. Liver Antioxidant Indices

The effects of *B. subtilis* ANSB060 on liver antioxidant indices of ducks exposed to aflatoxins are presented in Table 3. The result indicated that a low dose of aflatoxins in diets (Group M0) can decrease the activities of liver T-SOD and GSH-Px in ducks during the experimental period (*p* < 0.05); however, the presence of *B. subtilis* ANSB060 in some diets (Group M500, M1000 and M2000) exhibited a powerful capability of recovering T-SOD and GSH-Px activities in duck livers. The MDA level in liver was detected as the index of lipid peroxidation, and there were no significant effects of dietary aflatoxins and *B. subtilis* ANSB060 on MDA levels in duck livers in all groups (*p* > 0.05).

### 2.4. Aflatoxin Residues in Liver

The aflatoxin residues in the livers of ducks fed moldy diet naturally contaminated with 22.44 ± 2.46 μg/kg AFB_1_ with or without *B. subtilis* ANSB060 are given in Table 4. Aflatoxins were not detected in the livers of ducks consuming a normal diet (Group C0). The AFB_1_ and AFM_1_ residues in Group M0 were the highest, being 0.12 and 0.10 ng/g, respectively. *B. subtilis* ANSB060 in diets could degrade aflatoxins into nontoxic products in the duck intestinal tract such that AFB_1_ and AFM_1_ residues in the liver were decreased. The AFB_1_ residues was significantly decreased by 41.7%, 50.0%, and 58.3% respectively in Groups M500, M1000, and M2000 compared to those in Group M0 (*p* < 0.05). Similarly, the AFM_1_ residues were significantly reduced by 40.0% in Groups M500, M1000, and M2000, respectively. The AFB_2_, AFG_1_, and AFG_2_ residues in liver were not detected in all treatments.

## 3. Discussion

### 3.1. Growth Performance

Aflatoxins are mycotoxins produced by *Aspergillus* spp. that contaminate animal feed and human food. Aflatoxins can lead to growth reduction, immunity suppression, and oxidative damage, along with residues in animal tissues [9,32,33,34], and it has become a threat to human health. *B. subtilis* ANSB060 has shown a strong ability to detoxify aflatoxins in vitro; the degradation percentages of aflatoxins B_1_, M_1_, and G_1_ in this strain are 81.5%, 60%, and 80.7%, respectively [25]; meanwhile, their protective effects on the performance and antioxidant function in layers [26] and the reduced accumulation of aflatoxin residues in livers of broilers [27] have also been verified.

In this study, ducks fed diets contaminated with 22.44 ± 2.46 μg/kg of AFB_1_ show a tendency to decline in body weight, ADG, and feed efficiency compared with ones in the control group, illustrating a toxic effect of aflatoxins on the growth performance of ducks, which is consistent with previous research. A significant decrease in ADG and ADFI in ducks fed a diet containing 200 ppm AFB_1_ for three weeks were observed in the research conducted by Cheng et al. (2001) [35]. Meanwhile, Fowler et al. (2015) reported a reduced body weight (BW) from the first week to the third week in broilers fed aflatoxin-contaminated diets, and this reduction grew greater along with the increase of aflatoxin concentrations in diets from 0 to 1800 µg/kg; the percentages of body weight reduction ranged from 8.8% to 24.5% during Week 2, and from 15.6% to 41.3% during Week 3. Additionally, a negative effect of aflatoxins on the cumulative F:G ratio of broiler chicks was also shown [36]. However, the deleterious effect on the ADG of ducks in our study was not significant during the trial phase. This may be due to lower concentration of aflatoxins and the inadequate duration of the experiment. Previous results indicate that a decrease in ADG was due to anorexia, reluctance, and an inhibition of protein synthesis and lipogenesis [37,38,39]. This growth inhibition in ducks suggests that diets naturally contaminated with aflatoxins at a low level (22.44 ± 2.46 μg/kg) appear to have a toxic effect on the growth performance of ducks in vivo. This result indicates that aflatoxin degradation preparation mainly consisting of *B. subtilis* ANSB060 has a great ability to ameliorate the toxic effects of aflatoxins on the growth performance of ducks. The basic mechanism seems to be one in which the spores of *B. subtilis* ANSB060 can survive and colonize in an animal’s intestinal tract and secrete active substances to degrade aflatoxins in diets so that the adsorption of aflatoxins declines [27]. Some studies have suggested that microbial enzymes cleave the lactone ring of the AFB_1_ molecule in vitro to lower its toxicity [40,41]. Nevertheless, the specific biotransformation mechanism of *B. subtilis* ANSB060 detoxifying aflatoxins in the animal’s intestinal tract is unclear, and further studies should be performed with this strain [28].

### 3.2. Serum and Liver Antioxidant Indices

The inability to protect against reactive oxygen species (ROS) is the origin of oxidative stress. ROS are produced during the procedure of normal metabolism, and do much harm to the animal body. ROS mainly contain hydrogen peroxide (H_2_O_2_), a superoxide anion radical (O_2_^●^^−^), and a hydroxyl radical (^●^OH). ^●^OH is one of the most toxic oxygen-based radicals and wreaks havoc within cells, particularly with macromolecules. There is a dynamic equilibrium between oxidant and antioxidant systems and is vital for cell function, regulation, and adaptation to diverse growth conditions to remain healthy in animal bodies [42], and oxidant stress can be induced subsequently if the balance is broken down and the generation of ROS in a system exceed the system’s ability to neutralize and eliminate them [43]. Oxidant stress can cause biological membrane lipid oxidation, denaturation of intracellular protein and enzymes, DNA damage, pain, and ultimately various diseases. Enzymes such as SOD and GSH-Px are crucial components of the antioxidant system and play a key role in removing ROS and relieving oxidative damage [42]. MDA is the main product of lipid peroxidation that can bring about protein damage and inactivation of membrane-bound enzymes [44]; thus, the level of MDA can be an important index reflecting the body’s antioxidant ability [45].

One of our aims is to study the effects of adding *B. subtilis* ANSB060 to diets contaminated with a concentration of 22.44 ± 2.46 μg/kg aflatoxins on the antioxidant function in ducks. Our findings related to body antioxidant ability indicate that feeding diets contaminated with 22.44 ± 2.46 μg/kg aflatoxins can result in a significant decrease in T-SOD activity and an increase in the MDA level both in serum and the liver, while the activity of GSH-Px in duck livers is markedly decreased compared with that in the control group from Day 18 to Day 39. These results are in accordance with previous studies [46,47,48,49], demonstrating that lipid peroxidation took place in the ducks fed diet contaminated with aflatoxins.

While the activity of T-SOD is significantly increased and the MDA level is relatively decreased both in serum and liver, the activity of GSH-Px has a tendency to increase in serum but markedly increase in liver when ducks ingest aflatoxin-contaminated diets containing *B. subtilis* ANSB060 during the experiment compared with those fed no *B. subtilis* ANSB060 (M0). SOD and GSH-Px take part in lipid peroxidation by catalyzing the conversion of lipid hydroperoxide to hydroxy acids in the presence of GSH [50]. Liver is shown to be the major target organ for aflatoxins [51], and the increase in SOD and GSH-Px activities as well as the decrease in MDA level in serum and liver reflect that *B. subtilis* ANSB060 contributes to the inhibition of lipid peroxidation and relieve the harm produced by lipid peroxidation in ducks.

As a cause of liver degeneration and cancer, the elimination of these toxins from feedstuffs and foodstuffs bears great importance. In poultry, several nonnutritive adsorbents have been tested and have made some progress [46,52]; now, *B. subtilis* ANSB060 has been shown to have the capacity to inhibit lipid peroxidation, providing another biological method to alleviating oxidative damage to ducks caused by aflatoxins.

### 3.3. Aflatoxin Residues in Liver

The residue of aflatoxins and their metabolites in animal products have caused wide public concern. Liver is the main detoxifying organ that removes wastes and xenobiotics by metabolic conversion and biliary excretion [53]. Among the metabolites of AFB_1_, the toxicity of AFM_1_ in animals seems to be comparable to or slightly less than that of AFB_1_ [12]. In our study, low levels (0.12 and 0.10 ng/g) of AFB_1_ and AFM_1_ are retained in the livers of ducks fed the aflatoxin-contaminated diet for 21 days. Residue levels of AFB_1_ (0.05 and 0.13 μg/kg) and AFM_1_ (0.10 and 0.32 μg/kg) were also observed in the livers of broilers given diets contain 50 and 100 μg of AFB_1_/kg for 42 days [15]. AFB_1_ residue was found in the livers of laying hens fed 2.5 mg/kg AFB_1_ diet for four weeks [54]. Residue levels may be different because of the type of bird and diet, the concentrations of AFB_1_, and the duration, and enhanced tolerance to aflatoxins. In many countries, the maximum tolerance level of AFB_1_ in human food products is 2 ng/g. AFB_1_ residues in liver not only affect the performance and health of poultry, but also impair the health of the poultry product consumers as aflatoxins accumulate in edible parts of poultry, so it is necessary to control the quality of poultry products and analyze aflatoxin residues in different tissues of birds considering public health and safety. The results of our study show significantly decreased residue levels of AFB_1_- and AFM_1_-incorporated *B. subtilis* ANSB060 in ducks exposed to aflatoxins. The protective effects of *B. subtilis* ANSB060 from aflatoxins may be due to their specific biotransformation of aflatoxins in the intestinal tract, which leads to the reduction of aflatoxins absorbed by the intestinal tract and, consequently, a decrease in aflatoxin residues in the liver. However, the specific mechanism has not been tested, and more research should be done to determine this.

## 4. Conclusions

In conclusion, this study clearly indicates that aflatoxins in diets at a level of 22.44 μg/kg resulted in depressed growth performance and antioxidant capacity in liver and serum, as well as aflatoxin liver residues in ducks. Adding aflatoxin biodegradation preparation *B. subtilis* ANSB060 into moldy diets significantly recovers the growth performance of ducks and reduces the accumulation of aflatoxin residues in liver, effectively improving the antioxidant capacity to some extent. These results suggest that *B. subtilis* ANSB060 counteract the adverse effects of aflatoxins in animal diets, and thus is believed to be a very promising feed additive to detoxify aflatoxins in feed.

## 5. Materials and Methods

### 5.1. Ducks, Diets, and Management

One-day-old Cherry Valley ducks were obtained from a commercial hatchery and fed a commercial rearing diet for 17 days to adapt to the surroundings. On Day 18, 1500 male ducks were randomly assigned into 5 treatment groups with 6 replicates of 50 birds each. Five dietary treatment groups were composed as follows: Group C0 (the negative control), consisting of the basal diet with 60% normal maize; Group M0 (the positive control), containing 60% moldy maize taking the place of normal maize; and Groups M500, M1000, and M2000, containing an added 500 g/t, 1000 g/t and 2000 g/t of aflatoxin biodegradation preparation in the diet of Group M0, respectively. In this experiment, aflatoxin biodegradation preparation was mainly composed of industrially fermented and dried *B. subtilis* ANSB060 products via certain processing technology. The viable count of *B. subtilis* ANSB060 was more than 1 × 10^9^ CFU/g in the aflatoxin biodegradation preparation. The composition of the basal diet was presented in Table 5.

This study was approved by the Animal Care and Use Committee of the China Agricultural University (ethical approval code: CAU20131028-2; Date: 28 October 2013). All ducks were reared on floor in an environmentally controlled house equipped with central heating and temperature controllers. The size of barrier was 8 m × 2 m. Animals were cared for in accordance with the guidelines for the care and use of laboratory animals presented in the guide issued by the National Institute of Health and China’s Ministry of Agriculture. Body weight (BW), average daily gain (ADG), average daily feed intake (ADFI), and feed/gain ratio (F:G) were measured on a replicate basis at the end of the experiment (39 day old).

### 5.2. Analysis of Dietary Mycotoxin

The contents of mycotoxins (AFB_1_, AFB_2_, AFG_1_, AFG_2_, deoxynivalenol, zearalenone, and ochratoxin A) in feed ingredients and formulated diets in the study were determined using high performance liquid chromatography (HPLC) according to appropriate methods [55]. After these five diets were prepared, the final contents of mycotoxins for each diet were determined. The concentration of AFB_1_, AFB_2_, AFG_1_, AFG_2_, and zearalenone of the diets fluctuated at 22.44 ± 2.46 μg/kg, 6.69 ± 1.32 μg/kg, 1.65 ± 0.65 μg/kg, 0.00 μg/kg, and 5.20 ± 0.68 μg/kg in Groups M0, M500, M1000, and M2000, respectively. Only AFB_1_ was found in diet of Group C0, but the concentration of AFB_1_ was less than 0.20 μg/kg in this group.

### 5.3. Sampling of Antioxidant Indices and Measurements

At 39 day of age, 12 ducks (2 birds for each replicate) close to the average weight of treatment were slaughtered by dislocation of the neck vertebrae and bleeding after fasting for 12 h. Blood samples were collected by puncture of wing vein; after being centrifuged at 1000× *g* at 4 °C for 10 min, the serum was separated and transferred into 1.5 mL plastic centrifuge tubes for analysis of antioxidant indices including superoxide dismutase (SOD), glutathione peroxidase (GSH-Px) activity, and malondiadehyde (MDA) content.

The liver tissue samples were washed with ice-cold sterilized saline (0.85%), snap frozen in liquid nitrogen, and stored at −80 °C for further analysis of liver antioxidant indices. Liver tissue (1 g) was cut into small pieces and homogenized in ice-cold saline buffer (0.85%, pH = 7.4) (1:9, *w*/*v*) with an Ultra-Turrax (T8, IKA-labortechnik, Staufen, Germany) to form homogenates at a concentration of 0.1 g/mL for further analysis. Liver homogenates were centrifuged at 1000 *g* for 15 min at 4 °C, and the supernatants were collected. The supernatants were used for the assays of SOD and GSH-Px activity, and MDA content.

These parameters were determined with the clinical chemistry analyzer (Commercial Kit, Nanjing Jiancheng Bioengineering Institute, Nanjing, China) according to the manufacturer’s recommended procedure.

### 5.4. Analysis of Aflatoxin Residues in Liver

Livers samples from the pectoralis major of the right side of the duck were excised and frozen at −20 °C to analyze the residues of the aflatoxins. For this analysis, liver samples of six birds from each treatment (one for each replicate) were selected. Analysis of AFB_1_, AFB_2_, AFG_1_, AFG_2_, and AFM_1_ residues in the tissues was performed according to Fan et al. [27].

The ground defrosted liver sample (25 g), when 5 g of NaCl was added, was blended in 100 mL of methanol–water (80:20) for 3 min. Through a paper filter, an aliquot of 10 mL of filtrate was diluted with 40 mL of PBS/0.1% Tween-20 Wash Buffer and applied to an immunoaffinity column. Aflatoxins were eluted with 1.0 mL of methanol in a glass vial and dried near to dryness under a gentle stream of nitrogen and dissolved in a HPLC mobile phase. AFB_1_, AFB_2_, AFG_1_, and AFG_2_ were determined by the HPLC system (Shimadzu LC-10 AT) equipped with reverse phase column (Diamonsil, C18, 5 μm, 15 cm × 4.6 cm ID) and post-column photochemical derivatization (AURA, New York, NY, USA) and fluorescence monitor (Shimadzu RF-20A), with excitation at 360 nm and emission at 440 nm, with methanol/water (45:55) as the mobile phase, at a flow rate of 1 mL/min. For the determination of AFM_1_, HPLC was performed with a fluorescence monitor at 365 nm for excitation and 425 nm for emission, and water/acetonitrile/methanol (68:24:8) as the mobile phase at a flow rate of 1 mL/min.

### 5.5. Statistical Analyses

Data were analyzed using the GLM procedure of SAS software (version 9; SAS Institute, Inc., Cary, NC, USA). Duncan’s multiple range tests were used for multiple comparisons when the analysis indicated significant differences among treatments. All statements of statistical significance were based on probability (*p* < 0.05). All data were expressed as means ± SD.

## Figures and Tables

**Table 1:** Effects of *B. subtilis* ANSB060 on the growth performance of ducks fed moldy maize naturally contaminated with aflatoxins during the experiment.

Item	Initial BW, g	Final BW, g	ADG, g/bird d	ADFI, g/bird d	F:G, g/g
C0	759.75 ± 7.13	3159.73 ± 19.62	109.09 ± 0.78	235.28 ± 1.79	2.17 ± 0.02 ^a^
M0	758.15 ± 6.02	3120.93 ± 8.43	107.40 ± 0.53	234.28 ± 0.61	2.18 ± 0.01 ^a^
M500	754.92 ± 3.61	3193.96 ± 22.20	110.86 ± 1.05	237.23 ± 3.08	2.14 ± 0.02 ^a^
M1000	758.47 ± 5.26	3195.05 ± 11.78	110.75 ± 0.46	230.38 ± 2.21	2.08 ± 0.02 ^b^
M2000	759.60 ± 7.01	3186.97 ± 23.95	110.33 ± 1.31	237.32 ± 2.44	2.17 ± 0.03 ^a^
*p* Value	0.967	0.067	0.075	0.332	0.014

Each value is a mean ± SD from 6 replicates of 50 ducks each; ^a,b^ Means values with different superscripts in each column are significantly different (*p* < 0.05).

**Table 2:** The effects of *B. subtilis* ANSB060 on the serum antioxidant indexes of ducks fed moldy maize naturally contaminated with aflatoxins during the experiment.

Item	T-SOD, U/mL	GSH-Px, U/mL	MDA, nmol/mL
C0	112.41 ± 4.32 ^a,b^	1718.89 ± 141.66	2.55 ± 0.16
M0	107.36 ± 2.70 ^a^	1508.71 ± 184.24	2.83 ± 0.32
M500	121.50 ± 2.13 ^b^	1601.39 ± 81.31	2.62 ± 0.16
M1000	121.34 ± 4.12 ^b^	1818.50 ± 96.85	2.58 ± 0.28
M2000	120.30 ± 3.99 ^b^	1818.50 ± 264.37	2.45 ± 0.20
*p* Value	0.029	0.581	0.810

Each value is a mean ± SD from 6 replicates of 2 ducks each; ^a,b^ Means values with different superscripts in each column are significantly different (*p* < 0.05).

**Table 3:** The effects of *B. subtilis* ANSB060 on the liver antioxidant indices of ducks fed moldy maize naturally contaminated with aflatoxins during the experiment.

Item	T-SOD, U/mgprot	GSH-Px, U/mgprot	MDA, nmol/mgprot
C0	111.10 ± 5.73 ^c^	116.45 ± 6.12 ^a^	0.55 ± 0.05
M0	85.21 ± 3.44 ^a^	87.11 ± 5.28 ^b^	0.90 ± 0.29
M500	104.83 ± 3.50 ^b,c^	109.96 ± 3.31 ^a^	0.79 ± 0.08
M1000	98.37 ± 3.00 ^b^	106.58 ± 3.82 ^a^	0.56 ± 0.05
M2000	105.80 ± 3.92 ^b,c^	104.44 ± 5.86 ^a^	0.64 ± 0.06
*p*-value	0.001	0.007	0.398

Each value is a mean ± SD from 6 replicates of 2 ducks each; ^a^^–^^c^ Means values with different superscripts in each column are significantly different (*p* < 0.05).

**Table 4:** The effects of *B. subtilis* ANSB060 on aflatoxin residues (ng/g) in livers from ducks fed moldy maize naturally contaminated with aflatoxins during the experiment.

Item	AFB_1_	AFB_2_	AFG_1_	AFG_2_	AFM_1_
C0	ND *	ND	ND	ND	ND
M0	0.12 ± 0.01 ^a^	ND	ND	ND	0.10 ± 0.02 ^a^
M500	0.07 ± 0.01 ^b^	ND	ND	ND	0.06 ± 0.00 ^b^
M1000	0.06 ± 0.01 ^c^	ND	ND	ND	0.06 ± 0.01 ^b^
M2000	0.05 ± 0.01 ^d^	ND	ND	ND	0.05 ± 0.01 ^b^

Each value is a mean ± SD from 6 replicates of 1 duck each; ^a^^–^^c^ Means values with different superscripts in each column are significantly different (*p* < 0.05); * ND = Not detected.

**Table 5:** Basal diet formulations and nutritional contents.

Ingredients	Percentage (%)	Nutrition Component	Content
Maize	60.00	Crude protein, %	16.50
Soybean meal	13.64	Metabolizable energy, MJ/kg	12.39
Wheat	4.75	Calcium, %	1.00
Flour	3.50	Total phosphorus, %	0.67
Peanut meal	4.20	Available phosphorus, %	0.42
Zein meal	3.50	Methionine, %	0.46
Calcium hydrophosphate	1.36	Methionine + Cystine, %	0.76
Limestone	1.36	Lysine, %	0.96
Salt	0.32		
Soybean oil	1.77		
Rice bran	2.30		
Rice bran meal	2.00		
Premix ^1^	1.30		
Total	100.00		

^1^ Provided per kilogram of diet: vitamin A: 15,000 IU; cholecalciferol: 4000 IU; vitamin E: 40 IU; vitamin K3: 5.0 mg; thiamine: 3.0 mg; riboflavin: 3.0 mg; pyridoxine: 4.6 mg; vitamin B12: 0.08 mg; folic acid: 1.6 mg; niacin: 50 mg; pantothenic acid: 15 mg; choline chloride: 1000 mg; biotin: 0.25 mg; manganese: 100 mg; zinc: 100 mg; selenium: 0.3 mg; iodine: 0.3 mg; iron: 50 mg; copper: 15 mg.

## References

[1] Diener U.L., Cole R.J., Sanders T.H., Payne G.A., Lee L.S., Klich M.A. (1987). Epidemiology of Aflatoxin Formation by Aspergillus-flavus. Ann. Rev. Phytopathol..

[2] Goto T., Ito Y., Peterson S.W., Wicklow D.T. (1997). Mycotoxin producing ability of *Aspergillus tamarii*. Mycotoxins.

[3] Ito Y., Peterson S., Wicklow D., Goto T. (2001). *Aspergillus pseudotamarii*, a new aflatoxin producing species in *Aspergillus* section *Flavi*. Mycol. Res..

[4] Kurtzman C.P., Horn B.W., Hesseltine C.W. (1987). *Aspergillus-nomius*, a New Aflatoxin-producing Species Related to *Aspergillus-flavus* and *Aspergillus-tamarii*. J. Microbiol..

[5] Yiannikouris A., Jouany J. (2002). Mycotoxins in feeds and their fate in animals: A review. Anim. Res..

[6] Hussein H.S., Brasel J.M. (2001). Toxicity, metabolism, and impact of mycotoxins on humans and animals. Toxicology.

[7] Mishra H., Das C. (2003). A review on biological control and metabolism of aflatoxin. Crit. Rev. Food Sci..

[8] Groopman J.D., Hasler J.A., Trudel L.J., Pikul A., Donahue P.R., Wogan G.N. (1992). Molecular dosimetry in rat urine of aflatoxin-N7-guanine and other aflatoxin metabolites by multiple monoclonal antibody affinity chromatography and immunoaffinity/high performance liquid chromatography. Cancer Res..

[9] Rustemeyer S.M., Lamberson W.R., Ledoux D.R., Rottinghaus G.E., Shaw D.P., Cockrum R.R., Kessler K.L., Austin K.J., Cammack K.M. (2010). Effects of dietary aflatoxin on the health and performance of growing barrows. J. Anim. Sci..

[10] Dalvi R. (1986). An overview of aflatoxicosis of poultry: Its characteristics, prevention and reduction. Vet. Res. Commun..

[11] Kermanshahi H., Hazegh A.R., Afzali N. (2009). Effect of sodium bentonite in broiler chickens fed diets contaminated with aflatoxin B1. J. Anim. Vet. Adv..

[12] Magnoli A.P., Monge M.P., Miazzo R.D., Cavaglieri L.R., Magnoli C.E., Merkis C.I., Cristofolini A.L., Dalcero A.M., Chiacchiera S.M. (2011). Effect of low levels of aflatoxin B(1) on performance, biochemical parameters, and aflatoxin B(1) in broiler liver tissues in the presence of monensin and sodium bentonite. Poult. Sci..

[13] Berthiller F., Crews C., Dall’Asta C., Saeger S.D., Haesaert G., Karlovsky P., Oswald I.P., Seefelder W., Speijers G., Stroka J. (2013). Masked mycotoxins: A review. Mol. Nutr. Food Res..

[14] Ledoux D.R., Rottinghaus G.E., Bermudez A.J., Alonso-Debolt M. (1999). Efficacy of a hydrated sodium calcium aluminosilicate to ameliorate the toxic effects of aflatoxin in broiler chicks. Poult. Sci..

[15] Bintvihok A., Kositcharoenkul S. (2006). Effect of dietary calcium propionate on performance, hepatic enzyme activities and aflatoxin residues in broilers fed a diet containing low levels of aflatoxin B1. Toxicon.

[16] Yoshinari T., Noda Y., Yoda K., Sezaki H., Nagasawa H., Sakuda S. (2010). Inhibitory activity of blasticidin A, a strong aflatoxin production inhibitor, on protein synthesis of yeast: Selective inhibition of aflatoxin production by protein synthesis inhibitors. J. Antibiot..

[17] Karlovsky P. (1999). Biological detoxification of fungal toxins and its use in plant breeding, feed and food production. Nat. Toxins.

[18] Bata A., Lasztity R. (1999). Detoxification of mycotoxin-contaminated food and feed by microorganisms. Trends Food Sci. Technol..

[19] Wu Q., Jezkova A., Yuan Z., Pavlikova L., Dohnal V., Kuca K. (2009). Biological degradation of aflatoxins. Drug Metab. Rev..

[20] Kabak B., Dobson A.D.W., Var I. (2006). Strategies to prevent mycotoxin contamination of food and animal feed: A review. Crit. Rev. Food Sci..

[21] Taylor W.J., Draughon F.A. (2001). Nannocystis exedens: A potential biocompetitive agent against *Aspergillus flavus* and *Aspergillus parasiticus*. J. Food Protect..

[22] Liu D.L., Yao D.S., Liang R., Ma L., Cheng W.Q., Gu L.Q. (1998). Detoxification of aflatoxin B-1 by Enzymes Isolated from *Armillariella tabescens*. Food Chem. Toxicol..

[23] Teniola O.D., Addo P.A., Brost I.M., Farber P., Jany K.D., Alberts J.F., van Zyl W.H., Steyn P.S., Holzapfel W.H. (2005). Degradation of aflatoxin B-1 by cell-free extracts of *Rhodococcus erythropolis* and *Mycobacterium fluoranthenivorans* sp. nov DSM44556(T). Int. J. Food Microbiol..

[24] Zhao L.H., Guan S., Gao X., Ma Q.G., Lei Y.P., Bai X.M., Ji C. (2011). Preparation, purification and characteristics of an aflatoxin degradation enzyme from *Myxococcus fulvus* ANSM068. J. Appl. Mocrpbiol..

[25] Gao X., Ma Q., Zhao L., Lei Y., Shan Y., Ji C. (2011). Isolation of *Bacillus subtilis*: Screening for aflatoxins B1, M1, and G1 detoxification. Eur. Food Res. Technol..

[26] Ma Q.G., Gao X., Zhou T., Zhao L.H., Fan Y., Li X.Y., Lei Y.P., Ji C., Zhang J.Y. (2012). Protective effect of *Bacillus subtilis* ANSB060 on egg quality, biochemical and histopathological changes in layers exposed to aflatoxin B1. Poult. Sci..

[27] Fan Y., Zhao L., Ma Q., Li X., Shi H., Zhou T., Zhang J., Ji C. (2013). Effects of *Bacillus subtilis* ANSB060 on growth performance, meat quality and aflatoxin residues in broilers fed moldy peanut meal naturally contaminated with aflatoxins. Food Chem. Toxicol..

[28] Fan Y., Zhao L., Ji C., Li X., Jia R., Xi L., Zhang J., Ma Q. (2015). Protective Effects of *Bacillus subtilis* ANSB060 on Serum Biochemistry, Histopathological Changes and Antioxidant Enzyme Activities of Broilers Fed Moldy Peanut Meal Naturally Contaminated with Aflatoxins. Toxins.

[29] Anbiah S., Mohan C., Manohar B., Balachandram C. (2004). Chronic aflatoxin B-1 induced hepatopathy in ducks. Indian Vet. J..

[30] He J., Zhang K.Y., Chen D.W., Ding X.M., Feng G.D., Ao X. (2013). Effects of maize naturally contaminated with aflatoxin B1 on growth performance, blood profiles and hepatic histopathology in ducks. Liver Sci..

[31] Han X., Huang Q., Li W. (2008). Changes in growth performance, digestive enzyme activities and nutrient digestibility of cherry valley ducks in response to aflatoxin B-1 levels. Liver Sci..

[32] Yarru L.P., Settivari R.S., Gowda N.K., Antoniou E., Ledoux D.R., Rottinghaus G.E. (2009). Effects of turmeric (*Curcuma longa*) on the expression of hepatic genes associated with biotransformation, antioxidant, and immune systems in broiler chicks fed aflatoxin. Poult. Sci..

[33] Matur E., Ergul E., Akyazi I., Eraslan E., Inal G., Bilgic S., Demircan H. (2011). Effects of Saccharomyces cerevisiae extract on haematological parameters, immune function and the antioxidant defence system in breeder hens fed aflatoxin contaminated diets. Br. Poult. Sci..

[34] Li Y., Liu Y.H., Yang Z.B., Wan X.L., Chi F. (2012). The efficacy of clay enterosorbent to ameliorate the toxicity of aflatoxin B1 from contaminated corn (Zea mays) on hematology, serum biochemistry, and oxidative stress in ducklings. J. Appl. Anim. Res..

[35] Cheng Y.H., Shen T.F., Pang V.F., Chen B.J. (2001). Effects of aflatoxin and carotenoids on growth performance and immune response in mule ducklings. Comp. Biochem. Physiol..

[36] Fowler J., Li W., Bailey C. (2015). Effects of a Calcium Bentonite Clay in Diets Containing Aflatoxin when Measuring Liver Residues of Aflatoxin B1 in Starter Broiler Chicks. Toxins.

[37] Ghosh R.C., Chauhan H.V.S., Roy S. (1990). Immunosuppression in broilers under experimental aflatoxicosis. Br. Vet. J..

[38] Oguz H., Kurtoglu V. (2000). Effect of clinoptilolite on fattening performance of broiler chickens during experimental aflatoxicosis. Br. Poult. Sci..

[39] Oguz H., Kececi T., Birdane Y.O., Onder F., Kurtoglu V. (2000). Effect of clinoptilolite on serum biochemical and haematological characters of broiler chickens during experimental aflatoxicosis. Res. Vet. Sci..

[40] Guan S., Zhao L., Ma Q., Zhou T., Wang N., Hu X., Ji C. (2010). In Vitro Efficacy of *Myxococcus fulvus* ANSM068 to Biotransform Aflatoxin B1. Int. J. Mol. Sci..

[41] Liu D.L., Yao D.S., Liang Y.Q., Zhou T.H., Song Y.P., Zhao L., Ma L. (2001). Production, purification, and characterization of an intracellular aflatoxin-detoxifizyme from *Armillariella tabescens* (E-20). Food Chem. Toxicol..

[42] Yang J., Bai F., Zhang K., Bai S., Peng X., Ding X., Li Y., Zhang J., Zhao L. (2012). Effects of feeding corn naturally contaminated with aflatoxin B1 and B2 on hepatic functions of broilers. Poult. Sci..

[43] Fiers W., Beyaert R., Declercq W. (1999). More than one way to die: Apoptosis, necrosis and reactive oxygen damage. Oncogene.

[44] Coskun O., Kanter M., Korkmaz A. (2005). Quercetin, a flavonoid antioxidant, prevents and protects streptozotocin-induced oxidative stress and beta-cell damage in rat pancreas. Pharmacol. Res..

[45] Wills E.D. (1966). Mechanisms of Lipid Peroxide Formation in Animal Tissues. Biochem. J..

[46] Essiz D., Altintas L., Das Y.K. (2006). Effects of aflatoxin and various adsorbents on plasma malondialdehyde levels in quails. Bull. Vet. Inst. Pulway.

[47] Eraslan G., Akdogan M., Yarsan E., Sahindokuyucu F., Essiz D., Altintas L. (2005). The effects of aflatoxins on oxidative stress in broiler chickens. Turk. J. Vet. Anim. Sci..

[48] Guerre P., Larrieu G., Burgat V. (1999). Cytochrome P450 decreases are correlated to increased microsomal oxidative damage in rabbit liver and primary cultures of rabbit hepatocytes exposed to AFB1. Toxicol. Lett..

[49] Meki A., Esmail E., Hussein A. (2004). Caspase-3 and heat shock protein-70 in rat liver treated with aflatoxin B1: effect of melatonin. Toxincon.

[50] Preetha S.P., Kanniappan M., Selvakumar E., Nagaraj M., Varalakshmi P. (2006). Lupeol ameliorates aflatoxin B1-induced peroxidative hepatic damage in rats. Comp. Biochem. Physiol..

[51] Marin S., Ramos A.J., Cano-Sancho G., Sanchis V. (2013). Mycotoxins: Occurrence, toxicology, and exposure assessment. Food Chem. Toxicol..

[52] Ortatatli M., Oguz H., Hatipoglu F., Karaman M. (2005). Evaluation of pathological changes in broilers during chronic aflatoxin (50 and 100 ppb) and clinoptilolite exposure. Res. Vet. Sci..

[53] Taub R. (2004). Liver regeneration: From myth to mechanism. Nat. Rev. Mol. Cell Biol..

[54] Zaghini A., Martelli G., Roncada P. (2005). Mannanoligosaccharides and Aflatoxin B1 in Feed for Laying Hens: Effects on Egg Quality, Aflatoxins B1 and M1 Residues in Eggs, and Aflatoxin B1 Levels in Liver. Poult. Sci..

[55] Binder E.M., Tan L.M., Chin L.J., Handl J., Richard J. (2007). Worldwide occurrence of mycotoxins in commodities, feeds and feed ingredients. Anim. Feed Sci. Technol..

